# Comparative genomics of *Acinetobacter baumannii* and therapeutic bacteriophages from a patient undergoing phage therapy

**DOI:** 10.1038/s41467-022-31455-5

**Published:** 2022-06-30

**Authors:** Mei Liu, Adriana Hernandez-Morales, James Clark, Tram Le, Biswajit Biswas, Kimberly A. Bishop-Lilly, Matthew Henry, Javier Quinones, Logan J. Voegtly, Regina Z. Cer, Theron Hamilton, Robert T. Schooley, Scott Salka, Ry Young, Jason J. Gill

**Affiliations:** 1 Center for Phage Technology, Texas A&M University, College Station, TX USA; 2 Department of Biochemistry and Biophysics, Texas A&M University, College Station, TX USA; 3 Biological Defense Research Directorate, Naval Medical Research Center, 8400 Research Plaza, Fort Detrick, MD USA; 4 The Geneva Foundation, 917 Pacific Ave, Suite 600, Tacoma, WA USA; 5 Leidos, 11951 Freedom Dr., Reston, VA USA; 6 Naval Air Station, 6801 Roosevelt Blvd, Jacksonville, FL USA; 7 Department of Medicine, University of California, San Diego, La Jolla, CA USA; 8 AmpliPhi Biosciences (now Armata Pharmaceuticals), Marina Del Rey, CA USA; 9 Department of Animal Science, Texas A&M University, College Station, TX USA; 10 Present Address: MilliporeSigma, 14920 Broschart Rd, Rockville, MD USA

**Keywords:** Bacteriophages, Clinical microbiology, Antimicrobial resistance, Bacterial infection

## Abstract

In 2016, a 68-year-old patient with a disseminated multidrug-resistant *Acinetobacter baumannii* infection was successfully treated using lytic bacteriophages. Here we report the genomes of the nine phages used for treatment and three strains of *A. baumannii* isolated prior to and during treatment. The phages used in the initial treatment are related, T4-like myophages. Analysis of 19 *A. baumannii* isolates collected before and during phage treatment shows that resistance to the T4-like phages appeared two days following the start of treatment. We generate complete genomic sequences for three *A. baumannii* strains (TP1, TP2 and TP3) collected before and during treatment, supporting a clonal relationship. Furthermore, we use strain TP1 to select for increased resistance to five of the phages in vitro, and identify mutations that are also found in phage-insensitive isolates TP2 and TP3 (which evolved in vivo during phage treatment). These results support that in vitro investigations can produce results that are relevant to the in vivo environment.

## Abstract (web summary)

A patient with a multidrug-resistant bacterial infection was successfully treated in 2016 using phage therapy. Here, the authors sequence the genomes of the therapeutic phages and three bacterial strains isolated before and during treatment, and show that the same mutations conferring phage resistance are found in in vitro-generated mutants and in phage-insensitive strains isolated from the patient.

## Introduction

The Gram-negative bacterium *Acinetobacter baumannii* is recognized as one of the most important pathogens in healthcare-associated infections, particularly with ventilator-associated pneumonia and catheter associated infections^1–3^. This is especially true for carbapenem-resistant *A. baumannii*, which caused 8500 infections and 700 deaths in the U.S. in 2017 alone^4^. Several characteristics of this pathogen impact treatment regimens and outcomes, including the increased prevalence of multidrug-resistant (MDR) strains, environmental persistence due to its desiccation and disinfectant resistance, biofilm formation, and motility^5,6^. These results in hampered clinical intervention strategies and increased risks of reinfection and outbreaks^7^. As cases of resistant infections are more prevalent and very few new antibiotics are available, the use of bacteriophages (phages) to treat and/or control MDR infections is being reconsidered as an alternative strategy for therapeutic and prophylactic applications^8–10^.

In the modern era, the first published emergency intervention using phage in treating a systemic MDR *A. baumannii* infection in the US was the well-publicized “Patterson case” in 2016^11^. Clinical interventions using phage therapy to combat MDR bacterial infections have increased significantly in the past several years. Other well-known cases include the treatment of a chronic *Pseudomonas aeruginosa* infection in an aortic graft^12^, and an engineered phage approach to treat a cystic fibrosis patient with a recalcitrant *Mycobacterium abscessus* infection^13^. Successful phage treatment outcomes have reported in a number of case studies involving MDR *P. aeruginosa, Staphylococcus aureus*, and *Escherichia coli*^14^, and this field has recently been reviewed in refs. ^15,16^. These case studies have been encouraging in terms of clinical outcome, but in-depth examination of the phage-host interaction during treatment and their implications for phage efficacy remains an area of active study.

In principle, the effectiveness of the phage treatment depends on the ability of phage to localize to and persist in the infected tissue and propagate lytically. During this process, both the phages and their bacterial hosts replicate and evolve, potentially reducing the ability of the phages to clear the infection. In the 2016 *A. baumannii* clinical intervention, emergence of phage resistance was reported 8 days following the initiation of phage treatment^11^. Due to the rapid response required for the 2016 clinical intervention, both the *A. baumannii* pathogen and the phages used in treatment were largely uncharacterized. Here we examine the genomes of the therapeutic phages, the emergence of phage resistance during treatment, and the in vivo evolution of the pathogen with complete genomes of three *A. baumannii* strains isolated before and during phage therapy. Genetic changes responsible for phage resistance developed in vivo are compared to resistance developed in vitro, and the implications for optimizing phage therapeutic interventions are discussed.

## Results and discussion

The clinical course of the *A. baumannii* infection and phage treatment, known as the “Patterson Case”, has been described previously^11^. Briefly, phage treatment was initiated with two-phage cocktails, each containing four phages: cocktail ΦPC was administered into abdominal abscess cavities through existing percutaneous drains, and cocktail ΦIV was administered intravenously. Near the end of patient treatment, a ninth phage, AbTP3phi1, was isolated to target the phage-resistant *A. baumannii* strain TP3 that arose during treatment. Phage AbTP3phi1 was administered intravenously in a two-phage cocktail (ΦIVB) in combination with one phage from cocktail ΦIV^11^. As a follow up study to this phage intervention case, we determined the genomes of the phages and also of the bacterial strains that were isolated during phage treatment.

### Genomic characterization of the treatment phages

All nine phages used in the treatment cocktails were sequenced to completion and their genomes are summarized in Table 1. Genome sequences of phages C2P12, C2P21 and C2P24 described as part of cocktail ΦPC^11^ were determined to be identical, so phage C2P24 was renamed as phage Maestro and is used as a representative of this group. The phages described here can be categorized into two broad groups: phages Maestro, AC4, AB-Navy1, AB-Navy4, AB-Navy71 and AB-Navy97 are large (165–169 kb) T4-like myophages, and phage AbTP3phi1 is a 42 kb Fri1-like podophage.

**Table 1:** Characteristics of phages included in three cocktails used for treatment of a disseminated *A. baumannii* infection.

Phage cocktail	Phage name	Source	Propagation host	Morphology	Accession #	Genome size (bp)	ICTV taxonomic subfamily and genus	Closest classified relative in genus (nucleotide identity)
ΦPC (via intracavitary route)	Maestro^a^	CPT TAMU	TP1	Myophage	MT949699	169,176	*Twarogvirinae, Hadassahvirus*	AbTZA1 (NC_049445), 91.4%
	AC4	Ampliphi Biosciences	TP1	Myophage	OL770262	168,186	*Twarogvirinae, Lazarusvirus*	vB_ApiM_fHyAci03 (NC_049438), 93.7%
ΦIV (via intravenous route)	AB-Navy1	NMRC	TP1	Myophage	OL770258	166,113	*Twarogvirinae, Lazarusvirus*	AM101 (NC_049511), 94.9%
	AB-Navy4	NMRC	TP1	Myophage	OL770259	166,964	*Twarogvirinae, Lazarusvirus*	vB_ApiM_fHyAci03 (NC_049438); 94.8%
	AB-Navy71^b^	DSMZ	TP1	Myophage	OL770260	166,382	*Twarogvirinae, Hadassahvirus*	phage AbTZA1 (NC_049445); 96.5%
	AB-Navy97^c^	NMRC	TP1	Myophage	OL770261	165,480	*Twarogvirinae, Lazarusvirus*	AM101 (NC_049511); 95.8%
ΦIVB (via intravenous route)	AB-Navy97^c^	NMRC	TP1	Myophage	OL770261	165,480	*Twarogvirinae, Lazarusvirus*	AM101 (NC_049511); 95.8%
	AbTP3phi1	NMRC	TP3	Podophage	OL770263	42,093	*Beijerinckvirinae, Friunavirus*	vB_AbaP_B09_Aci08 (NC_048081), 84.0%

*CPT TAMU* Center for Phage Technology, Texas A&M University, *NMRC* Naval Medical Research Center, *DSMZ* Leibniz Institute DSMZ.

^a^Maestro is the representative of the three identical phages, C1P12, C2P21, and C2P24.

^b^AB-Navy71 was purchased from DSMZ under the name vB-GEC_Ab-M-G7.

^c^AB-Navy97 was used in both ΦIV and ΦIVB cocktails.

**Fig. 1:**
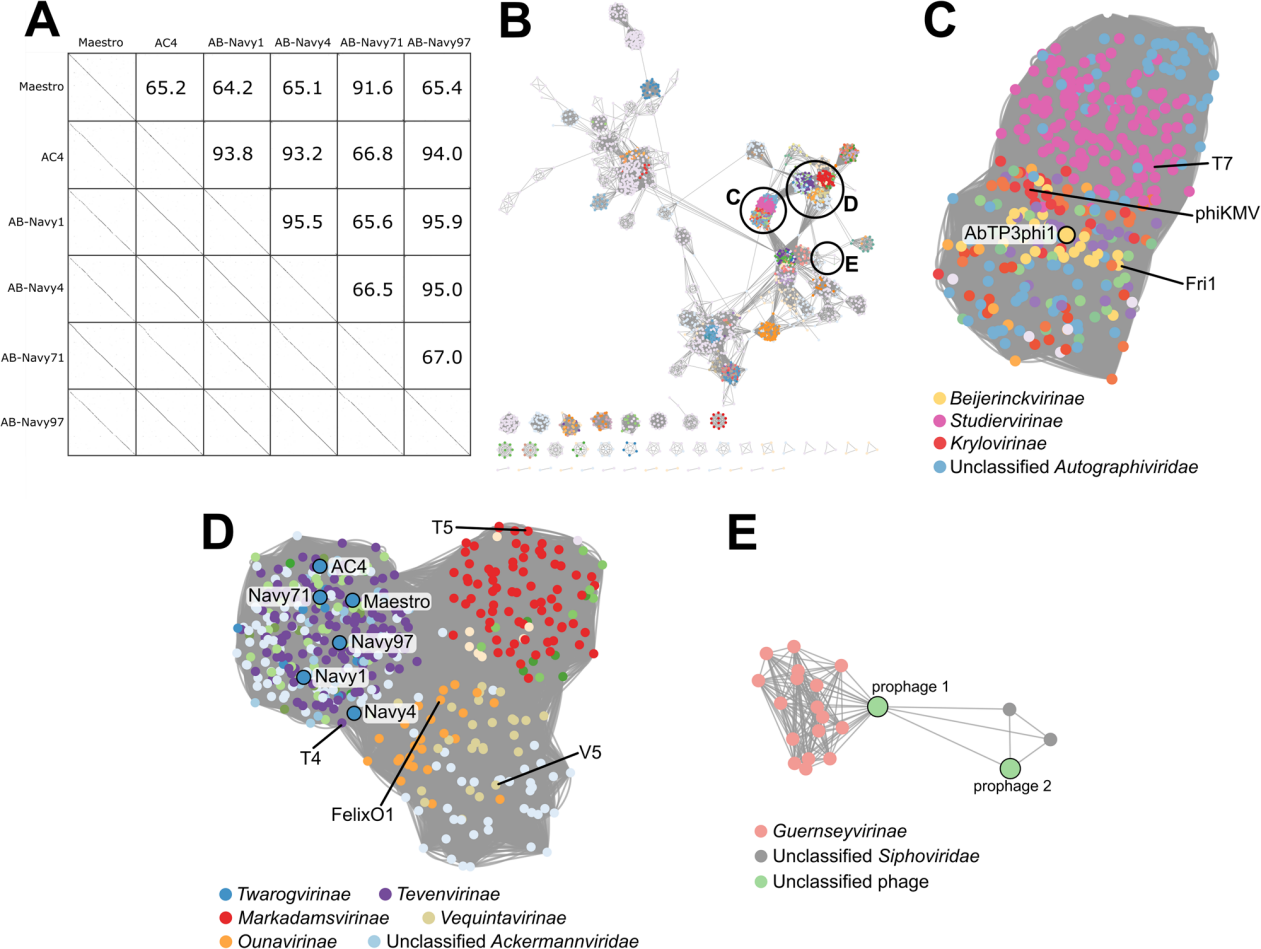
DNA and protein-based relationships of the seven treatment phages. **A** DNA sequence relatedness of six T4-like myophages, showing pairwise percent DNA sequence identities as determined by ProgressiveMauve (upper section) and DNA dotplots visually representing DNA sequence alignments between phages (lower section). **B** Protein sequence-based relationships of 2834 *Caudoviricetes* phages representing all species in the ICTV taxonomy, plus the seven treatment phages and two prophages identified in strains TP1, TP2, and TP3. Unclustered singletons (17 in total) are removed from the visualization. Distinct colors are assigned at the Subfamily level; if no Subfamily was assigned, color is assigned at the Family level. Circled clusters are enlarged in **C**–**E** as labeled. **C** Enlarged cluster representing the *Autographiviridae*. Nodes are colored based on their Subfamily membership, with the legend identifying prominent clades; the node representing phage AbTP3phi1 is outlined in black, and nodes representing clade-founding phages T7, phiKMV and Fri1 are labeled. **D** Enlarged cluster containing the T4-like subfamilies, including the *Twarogvirinae*; this large cluster is also linked to the T5-like *Markadamsvirinae* and clusters of diverse myophages including the V5-like *Vequintaviridae* and FelixO1-like *Ounavirinae*. Nodes are colored based on their Subfamily membership with the legend identifying prominent clades. Nodes representing the six treatment myophages are outlined in black, and nodes representing clade-founding phages T4, T5, FelixO1 and V5 are labeled. **E** Enlarged cluster containing the two prophage elements identified in strains TP1, TP2 and TP3. These prophages are not closely related to other classified phages, with the 52 kb prophage 1 distantly linked to the *Guernseyvirinae*, and the 42 kb prophage 2 related to two other unclassified siphophages.

The six myophages can be subdivided into two clusters, with Maestro and AB-Navy71 sharing 91.6% identity and phages AC4, AB-Navy1, AB-Navy4, AB-Navy97 sharing from 93.2% to 95.5 identity (Fig. 1A). Based on their sequence relationships to other *A. baumannii* phages and the criteria of the International Committee on Taxonomy of Viruses (ICTV)^17^, Maestro forms a new species in the genus *Hadassahvirus*, and phages AC4 and AB-Navy4 each form new species in the genus *Lazarusvirus* (Table 1). Phages AB-Navy1, AB-Navy71 and AB-Navy97 can be assigned to species in the genus *Hadassahvirus* or *Lazarusvirus* (Table 1). All of these phages are members of the Subfamily *Twarogvirinae* within the Family *Straboviridae*, which also contains the broadly-defined T4-like myophages, including the coliphage T4 itself. The 42 kb podophage AbTP3phi1 is classified as a new species within the genus *Friunavirus* of the Subfamily *Beijernickvirinae* (Table 1). It shares 82–89% overall DNA identity, as well as genome synteny, with previously described *Acinetobacter* podophages, including IME200 (NC_028987), vB_AbaP_AS11 (NC_041915), Fri1 (KR149290)^18^ and Aci08 (NC_048081).

Phage taxonomy is rapidly evolving, with multiple major recent and proposed revisions to the organization of phage taxa based on genomic relationships^19^. A recent global analysis of 134 *Acinetobacter* phage genomes placed these into eight major clusters and 38 sub-clusters, which include five proposed new subfamilies and 30 new genera^20^. The most abundant groups in this analysis are members of the *Twarogvirinae* and the *Beijernickvirinae*, with the vast majority of the latter (45/49 phages) falling into a single genus, the *Friunavirus*. A comparison of the seven treatment phages to a database constructed from species representatives of the *Caudoviricetes* (tailed dsDNA phages) in the ICTV taxonomy (Fig. 1B) shows higher-order relationships and diversity of these phages. This analysis produced a major grouping representing the *Autographiviridae*, which contains AbTP3phi1 (Fig. 1C) and a grouping containing the *Twarogvirinae* and *Tevenvirinae*, containing the six treatment myophages (Fig. 1D). This analysis placed the five current ICTV *Twarogvirinae* genera into three sequence-based viral clusters, with the treatment phages placed in a viral cluster with the other members of the genera *Hadassahvirus* and *Lazarusvirus*. Likewise, all members of the genus *Friunavirus* were placed into a single viral cluster with AbTP3phi1.

The genome of Maestro is presented as a representative for this group of *Acinetobacter* myophages (Supplementary Fig. 1). Maestro has a complete genome size of 169,176 bp and a GC content of 36.6%. Seven tRNA genes were identified, including one that appears to specify an amber codon. Genes encoding phage integrases or proteins associated with bacterial virulence were not detected. A conserved core of 95 genes encoding proteins with direct identity to coliphage T4 (BLASTp, E < 10^−5^) were identified, clustered in several regions of the genome. These include genes encoding structural proteins and proteins involved in DNA nucleotide metabolism and replication. Proteins involved in transcriptional regulation in phage T4 were found to have homologs in Maestro, which suggests Maestro follows a T4-like program of gene expression, with positive control of early, middle and late transcripts^21^. The holin and endolysin lysis genes in Maestro are similarly located as in T4 and have high primary structure similarity, indicating that the first two steps in lysis, the permeabilization of the inner membrane and the degradation of the cell wall are effected the same way^22^. The third step, disruption of the outer membrane, is accomplished in most dsDNA phages by spanin proteins^23^. No candidate spanins were detected in the Maestro genome, indicating that Maestro, like some other *Acinetobacter* phages, uses a different mechanism for OM disruption^23,24^. Homologs of the phage T4 RI and RIII antiholins were identified in the Maestro genome, indicating this phage has the ability to undergo T4-like lysis inhibition^25^. The effects of lysis inhibition in therapeutic interventions are not known, but superinfection-induced lysis inhibition delays lysis time and increases burst size in vitro and could affect in vivo phage proliferation at the site of therapeutic application. An analysis of 16 *Twarogvirinae* species representatives including Maestro, AB-Navy4 and AB-Navy97 by CoreGenes^26^ showed that 129 genes are conserved in this group, which includes the major DNA metabolism and structural functions, and a set of 41 hypothetical proteins with no identified function (Supplementary Fig. 1).

**Fig. 2:**
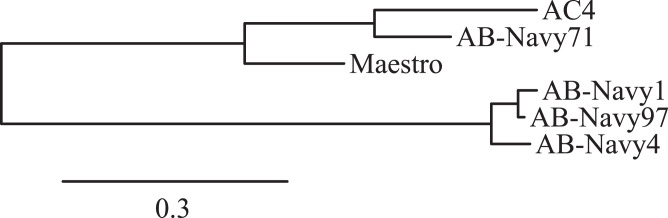
Phylogenetic tree of the long tail fiber protein sequences of the myophages used in phage treatment. The tail fibers of phages Maestro, AC4, and AB-Navy71 form one clade and the fibers of AB-Navy1, AB-Navy4 and AB-Navy97 form another.

During the infection process of phage T4, the long tail fibers (LTFs) bind to the phage’s receptor on the cell surface. In T4, the LTF is comprised of Gp34, Gp35, Gp36 and Gp37, which form the proximal LTF, two joints, and distal LTF, respectively; the distal LTF contains the phage receptor-binding function in its C-terminal domain^27,28^. The distal domains of the myophage LTFs, containing the predicted receptor-binding domains, were compared by multiple sequence alignment (Supplementary Fig. 2) and construction of a neighbor-joining tree to determine their relationships (Fig. 2). This analysis showed the myophages used in the cocktails had two different types of tail fibers, with Maestro, AC4, and Navy71 belonging to one group, and Navy1, Navy4, and Navy97 belong to the other cluster (Fig. 2). This finding correlates with the phage resistance patterns observed in *A. baumannii* strains isolated from the patient before and during phage treatment (Table 2). Strains resistant to phage AC4 were also resistant to phage Maestro and AB-Navy71, but the same strains were still partially sensitive to AB-Navy1, AB-Navy4, and AB-Navy97. Six days after the start of treatment, resistance to AB-Navy1, AB-Navy4 and AB-Navy97 was observed simultaneously. The closer relationship of the AC4 tail fiber to Maestro and AB-Navy71 likely represents a horizontal gene transfer event, as AC4 is overall more closely related to phages AB-Navy1, AB-Navy4 and AB-Navy97 (Fig. 1), and this points out a limitation of using whole-genome comparisons to predict the behavior of individual phages.

**Table 2:** Phage sensitivity of *A. baumannii* isolated from the patient before and during phage therapy.

Bacterial strains			Phages used for treatment						
Strain name	Date of isolation	Isolation source	Maestro	AC4	AB- Navy1	AB- Navy4	AB- Navy71	AB- Navy97	AbTP3phi1
TP1	02/10/2016		5 × 10^8^	6 × 10^10^	6 × 10^9^	5 × 10^9^	3 × 10^10^	2 × 10^8^	1 × 10^10^
AB-SD2	03/14/2016		6 × 10^8^	3 × 10^10^	5 × 10^9^	6 × 10^9^	8 × 10^9^	1 × 10^8^	ND
Start of phage therapy: March 15th									
AB-SD4	03/17/2016	Abscess-Drain 3	R	R	Z	Z	R	Z	3 × 10^9^
AB-2392	03/17/2016		R	R	Z	Z	R	1 × 10^5^	3 × 10^9^
Ab-2399	03/17/2016	Abscess-Drain 3	R	R	Z	Z	R	Z	3 × 10^9^
AB-SD1	03/19/2016	Abscess-Drain 5	R	R	1 × 10^5^	1 × 10^6^	R	2 × 10^4^	5 × 10^9^
AB-SD3	03/19/2016	Abscess-Drain 3	R	R	1 × 10^5^	Z	R	1 × 10^5^	3 × 10^9^
AB-2755	03/19/2016	Abscess-Drain 1	R	R	1 × 10^4^	1 × 10^4^	R	1 × 10^6^	3 × 10^9^
TP2	03/21/2016	Drainage 1	R	R	1 × 10^5^	1 × 10^5^	R	R	2 × 10^8^
AB-SD5	03/21/2016	Bronchial wash	R	R	R	R	R	R	2 × 10^9^
TP3	03/23/2016	Drain 1	R	Z	Z	Z	R	Z	1 × 10^9^
AB-0280	03/31/2016		R	R	R	R	R	R	4 × 10^9^
AB-1485	05/09/2016	Drain 3	R	R	R	R	R	R	1 × 10^7^
AB-3804	05/09/2016	Drain 1	R	R	R	R	R	R	1 × 10^7^
AB-3819	05/09/2016	Drain 3	R	R	R	R	R	R	1 × 10^7^
AB-3847	05/09/2016	Drain 5	R	R	R	R	R	R	1 × 10^7^
AB-0214	05/16/2016	Drain 5	R	R	R	R	R	R	2 × 10^7^
AB-0779	05/16/2016	Drain 1	R	R	R	R	R	R	1 × 10^9^
AB-0780	05/16/2016		R	R	R	R	R	R	1 × 10^9^

Phages were titered on strain TP1 and on lawns of the isolates obtained during the course of phage therapy. At high phage concentrations (>10^7^ PFU/ml), some phages were able to form clearing zones (denoted as Z) but no individual plaques were observed at lower dilutions. Phage resistance (no observable clearing at any phage concentration) is designated as R. All assays were performed in triplicate.

The podophage AbTP3phi1 shows conserved protein content and synteny with other members of the *Friunavirus* genus, which is part of the larger group *Autographiviridae* that also includes the well-studied *E. coli* podophage T7. The genome map of AbTP3phi1 is shown in Supplementary Fig. 3. As a conserved feature of this group of phages, a terminal repeat region of 396 bp was identified in AbTP3Φ1 genome by the PhageTerm tool^29^. Like T7, these phages possess relatively small genomes of ~40 kb and encode all proteins on one strand. Unlike T7, the gene encoding the RNA polymerase is located near the center of the genome, just upstream of the gene encoding the head-tail connector, an arrangement that is similar to that of phage phiKMV^30^. A CoreGenes analysis of 16 *Friunavirus* species representatives including AbTP3phi1 indicated that 29 out of 56 AbTP3phi1 protein-coding genes were conserved, which includes the DNA primase, helicase, ligase, polymerase, exo- and endonuclease, capsid, internal virion proteins, lysis proteins, the small and large terminase, and nine hypothetical proteins (Supplementary Fig. 3). Not conserved are a number of hypothetical proteins (mostly located near the left end of the genome) and the C-terminal portion of the tailspike, which contains capsular depolymerase activity^31^. Like other known *A. baumannii* Fri1-like podophages, tail spike protein of AbTP3phi1 contains a pectate lyase fold (PF12708) and thus uses the bacterial capsule as its receptor, degrading the bacterial exopolysaccharide as part of its infection process^20^. Strains TP1, TP2 and TP3 all encode KL116 capsule loci as determined by Kaptive^32^, thus the AbTP3phi1 depolymerase is presumed to be active against this capsule type. As with the myophages reported in this study, spanin proteins were not found in the genome of AbTP3phi1 nor in any other *A. baumannii* podophage genomes^24^, suggesting the presence of a novel strategy for disruption of the outer membrane in these phages.

### Phage and antibiotic sensitivity of *A. baumannii* strains isolated during treatment

During phage treatment, *A. baumannii* isolates were collected from the patient via various drains or bronchial washes. These strains were tested for their phage sensitivity via plaque assays. These showed that as early as 2 days after phage administration, the efficiency of all the phages in the first two cocktails (ΦPC and ΦIV) was reduced when tested against the bacterial strains isolated during treatment, evident by the decreased titers on those strains compared to the initial titers observed with TP1 (Table 2). In some cases, only a zone of clearing (but no individual plaques) was observed on the plates at high phage concentrations. Consistent with the myophage tail fiber protein sequence alignment (Fig. 2), host resistance to phages appeared earlier with Maestro, AC4, and Navy71 as a group, and later with phages Navy1, Navy4, and Navy97 as a group. In comparison, resistance to phage AbTP3phi1 was not observed in bacterial isolates collected throughout 2 months of phage treatment, although plating efficiencies of AbTP3phi1 varied by up to three orders of magnitude on strains collected during treatment (Table 2). The emergence of phage resistance early in phage treatment illustrates the potential benefits of well-characterized and rationally designed phage cocktails in treatment, which could be designed to mitigate the emergence of resistance. It also raises questions on the benefits of continued phage treatment beyond the first ~9 days, since all isolates collected after this time are fully resistant to the phage. While it is possible that the prolonged period of phage administration (over 60 days) was not required to produce the observed clinical outcome, other studies have shown that phage-insensitive mutants of *A. baumannii* exhibit reduced virulence^33–35^, a phenotype that has also been observed in other systems including *Staphylococcus aureus*^36^, *Klebsiella pneumoniae*^37^ and *P. aeruginosa*^38^. Thus, maintaining selection pressure for the phage-resistant phenotype may provide a benefit to continued treatment even after the pathogen has developed resistance to the treatment phage.

Some strains isolated throughout phage treatment were also tested for their antibiotic resistance profiles by traditional microtiter MIC (Supplementary Table 2). At the time TP1 was isolated from the patient, they were receiving a combination of fluconazole, azithromycin, colistin, and rifampin. Shortly after TP1 isolation, meropenem was added to treatment, and shortly after the beginning of phage administration the azithromycin, colistin, and rifampin were discontinued and minocycline was initiated. The meropenem, minocycline and fluconazole treatment was continued through the end of phage treatment^11^. In general, the antibiotic resistance profiles of all strains isolated during the course of phage therapy remained consistent, indicating that phage therapy did not have a major impact on antibiotic resistance of the pathogen in this case. Although an initial report indicated resistance to colistin and tigecycline prior to the start of phage therapy^11^, sensitivity to colistin and tigecycline (in the range of 2–8 ug/ml) was observed in strains isolated ~7 weeks after the start of phage therapy (collected on May 9, 2016). While sensitive to colistin and tigecycline, these strains were resistant to minocycline. We previously reported on a potential synergistic in vitro activity between phage cocktail and minocycline (used at sub-inhibitory concentrations of 0.25 ug/ml) in inhibiting bacterial growth^11^. However, such results were obtained using strain TP3, and TP3 was not tested for its sensitivity to minocycline, colistin, or tigecycline in these MIC assays. Increased antibiotic sensitivity has been associated with phage resistance in organisms such as *A. baumannii*^34,35^ and *P. aeruginosa*^39^. However, some studies have observed increased antibiotic resistance in phage-resistant mutants^40,41^, indicating increased sensitivity to antibiotics is not a universal outcome from phage resistance and is probably dependent on the host, drug, phage, and nature of the resistance mutation. While fitness costs can be associated with phage resistance, the effects of resistance mutations can be pleiotropic with phenotypes that are not always easily predictable^42^.

**Fig. 3:**
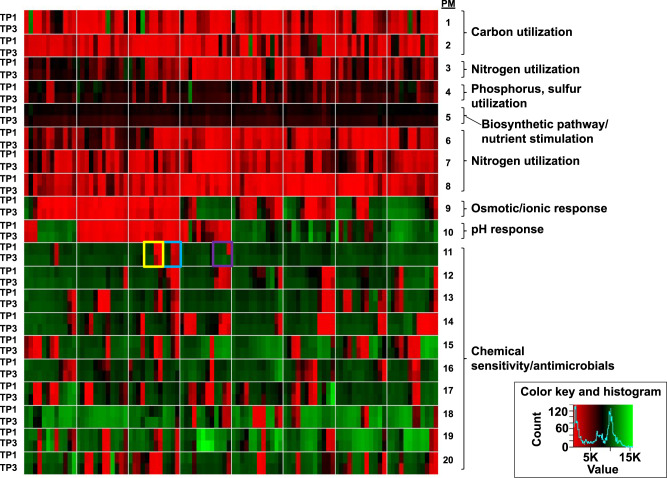
Phenotypic profiling of strains TP1 and TP3. Each row represents a bacterial isolate (TP1 or TP3) in one phenotype panel (PM01–PM20), and each column represents a specific condition (wells A01-H12) within each panel, as shown in Supplementary Data 1. Effects on bacterial metabolic activity are indicated by color, with red representing growth inhibition, black representing intermediate growth and green representing growth promotion. Yellow box: colistin. Light blue box: minocycline. Purple box: nafcillin. Results are calculated using the area under the curve for 48 h of growth and are presented as the average of three replicates per strain.

To more fully delineate the phenotypic differences between TP1 and TP3, BioLog phenotypic microarray (PM) profiling was conducted using PM 1–20 (Fig. 3 and Supplementary Data 1). As expected given the clonal nature of the isolates, the PM demonstrated very consistent phenotypes in terms of carbon, nitrogen, phosphorus and sulfur utilization; biosynthetic pathways and nutrient stimulation; osmotic/ionic response; and pH response; as well as very consistent phenotypes in the chemical sensitivity assays (Fig. 3). The phenotypic profiling results show that growth of both isolates TP1 and TP3 could be inhibited by colistin or minocycline at higher concentrations (Fig. 3, yellow box and light blue box, respectively); tigecycline sensitivity is not included in the phenotype microarray (PM) panel. Isolate TP3 was found to be completely resistant to nafcillin in this assay, whereas TP1 was sensitive (Fig. 3, purple box).

### Characterization of *A. baumannii* strains TP1, TP2, and TP3 isolated before and during phage therapy

**Fig. 4:**
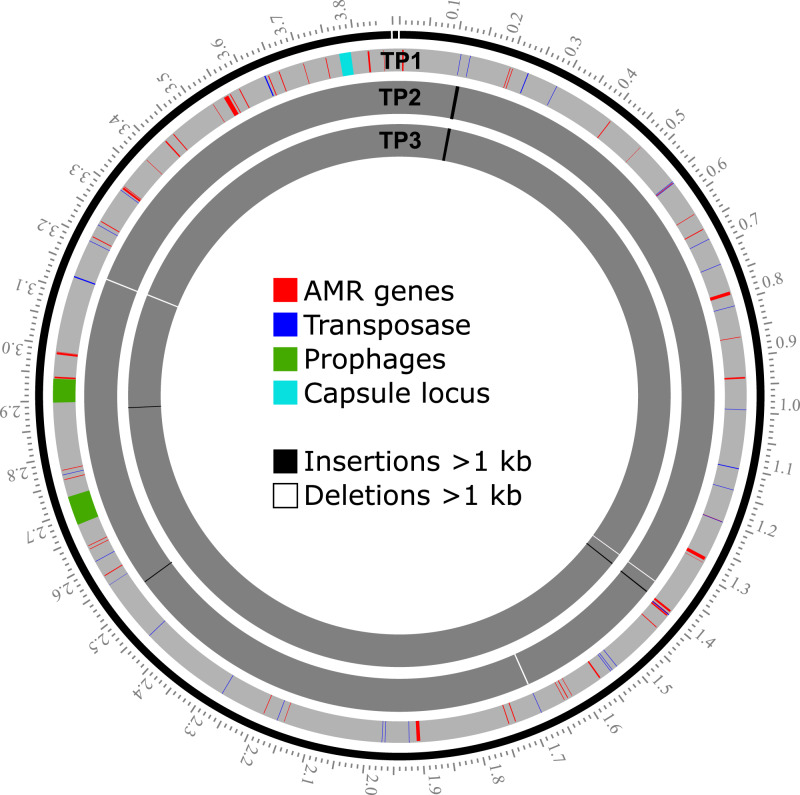
Locations of AMR genes, transposases, prophages, and capsule locus in the TP1 genome, and large insertion and deletions (>1 kb) in TP2 and TP3, in reference to TP1. Each gray band represents a bacterial chromosome as labeled at the replication origin. The scale on the outer ring represents DNA coordinates in Mb. The locations of AMR genes, transposases, prophages and the capsule locus are indicated for TP1 only. Major insertions or deletions (>1 kb) are indicated in the TP2 and TP3 chromosomes. Of note, TP2 and TP3 contain a novel 6.7 kb insertion element at the ~0.11 Mb position that is not present in TP1, indicating horizontal acquisition during infection.

Three *A. baumannii* isolates, TP1, TP2, and TP3, were sequenced to closure using a combination of short-read (Illumina) and long-read (Nanopore) sequencing to investigate pathogen evolution during the course of phage treatment. Sequencing to closure allows for tracking of the number and position of mobile DNA elements that are often not assembled into larger contigs if the genomes are only sequenced with short-read sequencing. Strain TP1 was isolated prior to the start of phage treatment and was the clinical isolate used to determine phage sensitivity and conduct environmental phage hunts for assembly of therapeutic phage cocktails^11^. Strains TP2 and TP3 were isolated 6 days and 8 days after the start of phage treatment. All three strains were found to contain a single 3.9 Mb chromosome and a single 8.7 kb plasmid (Table 3). Some variation was observed in bacterial chromosome length between strains but the plasmids contained in each strain were identical, and it is clear that these three isolates represent the evolution of strains from a common ancestor over time rather than a succession invasion by different strains. Analysis of the genomes in pubMLST^43^ identified all three isolates as sequence type 570 (Pasteur) and analysis in Kaptive^44^ identified a 20.5 kb region (base position 3,774,031–3,794,556 in the TP1 genome) containing 17 genes encoding a predicted capsule type of K116 (Supplementary Table 3). Consistent with the broad antibiotic resistance observed in these isolates, 32 (TP1) and 35 (TP2, TP3) antibiotic resistance genes (ARGs) were identified based on searches against the CARD 2021 database^45^ (Supplementary Data 2). The 8.7 kb plasmid contained in TP1, TP2 and TP3 does not encode any identifiable AMR genes, and is identical to plasmids carried in many other *A. baumannii* strains deposited in NCBI. Few SNPs and indels were observed between these isolates, including 2–3 large (>1 kb) insertions or deletions associated with the movement of mobile DNA elements. Summaries of the genomes and changes observed in strains TP2 and TP3 (relative to TP1) are summarized in Table 3, and the locations of AMR genes, transposases, prophages, capsule locus in TP1 genome, and large insertion and deletions (>1 kb) in TP2 and TP3, in reference to TP1, are illustrated in Fig. 4. In reference to TP1, detailed sequence changes, associated coordinates and genes affected in TP2 and TP3 are listed in Supplementary Tables 4 and 5, respectively.

**Table 3:** Summary of genome features and genetic changes observed in *A. baumannii* strains TP2 and TP3 relative to TP1.

Feature	*A. baumannii* strain		
	TP1	TP2	TP3
Isolation date	02/10/2016	03/21/2016	03/23/2016
Isolation source	Unknown	Drain 1	Drain 1
NCBI accession	CP056784, CP056785	CP060011, CP060012	CP060013, CP060014
Complete genome size (bp)	3,864,540	3,870,560	3,871,732
Plasmid number/size (bp)	1 (8731)	1 (8731)	1 (8731)
total coding genes	3556	3564	3564
Complete prophage elements	2	2	2
Large insertions^a^ (>1 kb)	n/a	3	3
Large deletions^a^ (>1 kb)	n/a	3	2
AMR genes	32	35	35

^a^Relative to strain TP1.

The most notable change in TP2 and TP3 is the acquisition of a novel 6673 bp insertion sequence, inserted in a position adjacent to an existing IS3-like transposase at position 111,357 of the TP1 genome (Fig. 4 and Supplementary Tables 4 and 5). This acquired 6.7 kb element is not native to TP1 and represents an acquisition of new DNA by horizontal gene transfer that occurred during the course of infection, and is most likely the result of DNA acquisition mechanisms unrelated to phage treatment. *A. baumannii* is known for its ability to rapidly acquire mobile DNA elements in the environment via conjugation and natural competence^46,47^, and to vary surface molecules through horizontal gene transfer^48^. T4-like phages like those used in treatment are generally poor transducers. In phage T4, multiple defects in *ndd*, *denB*, *42* and *alc* are required for transduction to occur^49^, and these genes are all conserved in the cocktail myophages reported in this study. In addition, transduction requires the phage to be able to productively infect the donor of the acquired DNA, which was likely to have been a different bacterial species and thus insensitive to the phages used. BLASTn searches of this sequence identified identical or nearly identical sequences in other Gram-negative bacterial genomes or plasmids, including *A. baumannii* (CP038644), *Klebsiella pneumoniae* (LR697132), *E. coli* (CP020524), and *Citrobacter freundii* (KP770032). This inserted sequence encodes a number of significant additional antibiotic resistance determinants, including a predicted aminoglycoside O-phosphotransferase (IPR002575), an NDM-1-like metallo-beta-lactamase (CD16300, IPR001279), and a CutA-like protein that may be involved in metal tolerance (IPR004323). The inserted aminoglycoside O-phosphotransferase (CARD ARO:3003687) is relatively rare in *A. baumannii*, found in 1.43% of *A. baumannii* chromosomes and 0.47% of *A. baumannii* plasmids, as reported by the CARD Resistance Gene Identifier. The prevalence of the inserted NDM-1-like metallo-beta-lactamase (CARD ARO:3000589) is 5.94% of *A. baumannii* genomes and 0.6% of *A. baumannii* plasmids.

Other than the 6.7 kb insertion described above, all other major variations in the TP2 and TP3 genomes can be attributed to deletion or transposition of elements present in the TP1 genome (Supplementary Tables 4 and 5). Another 1886 bp insertion sequence was identified in TP2 and TP3 which introduces a second copy of the IS6-like transposase and an additional copy of an aminoglycoside O-phosphotransferase (IPR002575) which is also present in TP1 (locus HWQ22_16890). In this case, this insertion is a duplication of an existing AMR gene rather than the acquisition of foreign DNA. The presence of the new 6.7 kb element and the duplicated 1.9 kb element resulted in three extra ARGs in TP2 and TP3 (35 total predicted AMR genes) compared to TP1 (32 total predicted AMR genes) (Table 3 and Supplementary Data 2). This highlights the fact that bacterial pathogens do not exist as strictly clonal populations even in a single patient over time.

### Prophage elements in TP1, TP2, and TP3

Prophage analysis revealed two apparently complete prophage regions (52,563 bp and 42,762 bp in length, respectively) in TP1, TP2, and TP3 genomes that are likely to encode active prophages (Fig. 4). Phage *att* sites and conserved phage proteins (tail and tail tape measure protein, major head subunit and head morphogenesis protein, terminase large subunit, endolysin) were identified in both prophage regions; the coordinates of the prophages in the TP1 genome are provided in Supplementary Table 3. These two prophage regions are conserved in TP1, TP2, and TP3 and no sequence change was observed among the three strains. The 52 kb prophage 1 is highly conserved (with up to 100% nucleotide identity by BLASTn) in many other *A. baumannii* genomes, including that of ATCC 19606, which is one of the earliest available clinical isolates of *A. baumannii* dating to the 1940’s^50^. This prophage region shares limited similarity to cultured phages, with its closest relative being *Acinetobacter* phage Ab105-3phi (KT588073), with which it shares 49.4% nucleotide identity and 22 similar proteins. The 43 kb prophage region was found to be less conserved in other *A. baumannii* genomes, with the most closely related prophage element sharing only 69% overall sequence identity. This prophage region is ~46% related to *A. baumannii* phage 5W (MT349887), which also appears to be a temperate phage due to the presence of an integrase and LexA-like repressor. Other than 5W, this element is not closely related to any other cultured phages in the NCBI database, sharing no more than 10% nucleotide identity and no more than 8 proteins with other phages. Protein-based clustering of these elements (Fig. 1E) showed that they are only distantly related to other cultured phages, with the closest neighbors in the *Guernseyvirinae*. A recent analysis of prophage carriage in *A. baumannii* genomes suggests that intact prophages are relatively uncommon in this species (less than one per genome) and also highly diverse, indicating a large amount of unexplored diversity in temperate phage elements^51^.

### Characterization of phage-resistant mutants generated in vitro and the comparison to in vivo isolates

**Fig. 5:**
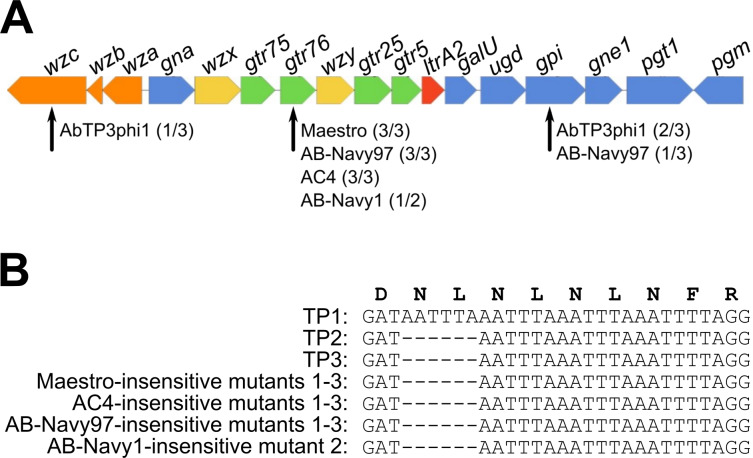
Comparison of the K loci of strains TP1, TP2 and TP3, and phage-resistant mutants generated in vitro. **A** Diagram of the KL116 capsule locus identified in strains TP1, TP2, and TP3 as predicted by Kaptive. Genes are represented by arrows oriented in the direction of transcription. Orange arrows represent genes involved in capsule export, yellow genes are involved in repeat unit processing, blue genes are involved in simple sugar biosynthesis, green genes encode glycotransferases and the red gene codes for the initiating transferase. All genes had 100% coverage and ranged from 90–100% identity to the KL116 type in the Kaptive database. Defective capsule locus genes identified in in vitro-generated phage-insensitive mutants of TP1 are indicated by black arrows; numbers in parentheses after each phage name indicate what proportion of phage-insensitive mutants contained a mutation in that gene. **B** Nucleotide alignment of the sequences showing the six nucleotide deletion in one of the glycosyltransferases (*gtr76*) found in multiple TP1 mutants resistant to the cocktail myophages.

Five phages selected from the phage cocktails (AC4, Maestro, AB-Navy1, AB-Navy97, AbTP3phi1) were used to select for phage-insensitive mutants in vitro using *A. baumannii* strain TP1 as host. Three independent mutants against phages AC4, Maestro, AB-Navy97, AbTP3phi1 were isolated, and two independent mutants against phage AB-Navy1 were isolated. After resequencing and mapping mutant reads to the reference TP1 genome, changes detected with quality scores greater than 100 were examined (Table 4). The majority of identified mutations were located in the bacterial KL116 capsule locus. The K116 capsule is comprised of a five-sugar repeating unit with a three-sugar backbone composed of Gal and GalNAc and a two-sugar side chain composed of Glc and GalNAc^52^. In all the mutants resistant to the myophages Maestro, AC4, AB-Navy97, a common 6-bp deletion was observed in a predicted capsular glycosyltransferase protein identified as Gtr76 by Kaptive (HWQ22_04225) (Fig. 5). Notably, these 6-bp deletions were also observed in isolates TP2 and TP3, which evolved in vivo during phage treatment and were insensitive or showed reduced sensitivity to all myophages tested (Table 2 and Supplementary Tables 4 and 5). These 6-bp deletions occurred in a region containing four copies of a tandem repeat sequence TAAATT (Fig. 5B), which probably is prone to mutation by strand slippage events during replication. These mutations result in the deletion of residue L243 and N244, resulting in the reduction of a predicted flexible linker between two α-helices in the C-terminus of the glycosyltransferase protein. This protein is predicted to participate in capsule synthesis by forming the β-D-GalNAc-(1 → 4)-D-Gal linkage of the side chain disaccharide to the trisaccharide backbone^52^, suggesting that this side chain plays a role in host recognition by these phages.

**Table 4:** Genetic changes identified in phage-resistant mutants of *A. baumannii* TP1 generated in vitro.

Phage mutant	Mutation type	Base position in TP1	Quality score	Gene function	Locus in TP1	Mutation
AB-Navy97 mutant 1	SNP	25961	225	Intergenic		T/C
	SNP	3779783	212	Glucose-6-phosphate isomerase	HWQ22_04190	W102I
	Deletion	3786204	217	Glycosyltransferase	HWQ22_04225	ΔL243-N244
	SNP	3813620	225	Sulfonate ABC transporter substrate-binding protein	HWQ22_04355	A123A (silent)
AB-Navy97 mutant 2	Deletion	3786204	217	Glycosyltransferase	HWQ22_04225	ΔL243-N244
AB-Navy97 mutant 3	Deletion	3786204	217	Glycosyltransferase	HWQ22_04225	ΔL243-N244
AB-Navy1 mutant 1	SNP	995279	175	Ornithine uptake porin CarO type 3	HWQ22_09280	W183am
AB-Navy1 mutant 2	Deletion	3786204	214	Glycosyltransferase	HWQ22_04225	ΔL243-N244
Maestro mutant 1	Deletion	3786204	214	Glycosyltransferase	HWQ22_04225	ΔL243-N244
Maestro mutant 2	Deletion	3786204	214	Glycosyltransferase	HWQ22_04225	ΔL243-N244
Maestro mutant 3	Insertion	1380798	115	Intergenic		9 bp insertion
	SNP	2895519	222	Intergenic		C/T
	Deletion	3786204	214	Glycosyltransferase	HWQ22_04225	ΔL243-N244
AC4 mutant 1	Deletion	3786204	198	Glycosyltransferase	HWQ22_04225	ΔL243-N244
AC4 mutant 2	Deletion	3786204	214	Glycosyltransferase	HWQ22_04225	ΔL243-N244
AC4 mutant 3	Deletion	3786204	209	Glycosyltransferase	HWQ22_04225	ΔL243-N244
AbTP3phi1 mutant 1	SNP	1696827	222	Intergenic		A/T
	SNP	3779456	222	Glucose-6-phosphate isomerase	HWQ22_04190	S211L
AbTP3phi1 mutant 2	SNP	3778893	222	Glucose-6-phosphate isomerase	HWQ22_04190	Q399oc
AbTP3phi1 mutant 3	Insertion	3794503	167	Polysaccharide biosynthesis tyrosine autokinase	HWQ22_04255	G715 frameshift

All changes are relative to the sequence of strain TP1.

In mutants selected for insensitivity to phage AB-Navy1, one mutant contained the same conserved 6 bp deletion identified in the other mutants, and one lacked this mutation but instead had a nonsense mutation (W183am) in *carO* (HWQ22_09280) (Table 4). CarO is a 29 kDa outer membrane transporter, loss of which has been associated with increased antibiotic resistance^53,54^. The role of CarO in phage sensitivity is not clear, but its truncation may lead to other cell wall defects that reduce sensitivity to this phage; truncations in CarO have been associated with reduced adherence and invasion in tissue culture and with reduced virulence in vivo^55^. This finding illustrates that defects in the capsule locus are not the only means by which TP1 may gain phage insensitivity. Notably, similar CarO defects were not observed in TP2 or TP3, which attained phage resistance in vivo.

In addition to the common 6 bp deletion in the Gtr76 glycosyltransferase and CarO mutation, the other mutations observed in the myophage-insensitive mutants are SNPs or small indels in non-coding regions or that result in missense or silent mutations in a predicted capsular glucose-6-phosphate isomerase Gpi (HWQ22_04190) and an ABC transporter, respectively (Table 4). However, these SNPs are not conserved in the in vitro mutants against myophages and were also not detected in in vivo isolates TP2 and TP3, suggesting that the defect observed in the Gtr76 glycosyltransferase is sufficient to confer insensitivity to the cocktail myophages in this strain.

Strain TP1 mutants resistant to the podophage AbTP3phi1 were also found to contain mutations in the capsule locus, but these mutations were confined to the genes encoding the glucose-6-phosphate isomerase Gpi and polysaccharide biosynthesis tyrosine autokinase Wzc (HWQ22_04255) (Table 4 and Fig. 5A). Loss of function in these genes is expected to result in loss of L-fructose-6-phosphate required for downstream production of capsule monomers and defects in capsule export, respectively^32^. This suggests that these mutants may exhibit more severe defects in K116 capsule expression, and that AbTP3phi1 requires the presence of the capsule backbone for successful infection.

Our results are consistent with the recently published work by Altamirano et al.^34^, where a frameshift in the glycosyltransferase and glucose-6-phosphate isomerase within the K locus were detected in two independent phage-resistant *A. baumannii* mutants. The consistency between our work and that study confirms the *A. baumannii* capsule locus being important for phage sensitivity. Both Gpi and glycosyltransferases are involved the biosynthesis of capsule K units, which are tightly packed repeating subunits consisting of 4 to 6 sugars^56^. The reason why one group of phages (our myophages, and the myophage ΦFG02 in ref. ^34^) selected primarily for defects in the Gtr glycosyltransferase but the other phages (our podophage AbTP3phi1 and myophage ΦCO01 in Altamirano et al.) selected for defects in Gpi is not entirely clear. These phages likely recognize different moieties of the bacterial capsule as their receptors, but it should be noted that many of the mutations associated with insensitivity observed in our study are not necessarily inactivating to the protein: the most common mutation in the capsule locus is a two-residue in-frame deletion in *gtr76* (Fig. 5B), and the other mutations are single-residue changes or nonsense/frameshift mutations relatively late in the reading frame. These mutations may modulate protein function rather than being inactivating.

Capsule is a known common requirement for *A. baumannii* phages, and defects in capsule synthesis have been shown to be responsible for phage resistance^34,57^. The presence of the same 6 bp deletion in the capsular glycosyltransferase gene *gtr76* of both the in vitro- and the in vivo-selected *A. baumannii* strains indicates that the same route to phage insensitivity may be followed by strain TP1 in both systems. Importantly, this demonstrates that laboratory in vitro investigations of bacterial selection and phage insensitivity can produce results that are relevant and predictive for the in vivo milieu of clinical treatment.

## Conclusions

In conclusion, this study provides detailed genomic information on the evolution of *A. baumannii* during the course of infection, showing that resistance to the therapeutic phages emerged early, and the acquisition of new mobile elements can occur during treatment. The majority of patient treatment was conducted with a mixture of T4-like myophages that are related to a clade of known *A. baumannii* myophages of the Subfamily *Twarogvirinae* (Fig. 1). The results of phage sequencing highlight the importance of thorough genomic analysis of phages prior to phage treatment in order to maximize treatment success and minimize effort and consumption of resources. While none of the phages used contain any detectable deleterious genes and appear to be strictly virulent, three of the phages used in cocktail ΦPC were found to be genetically identical, while the other phages used in the initial ΦPC and ΦIV cocktails are closely related and fall into only two groups based on tail fiber similarity (Fig. 2). This explains why *A. baumannii* isolates collected as soon as 2 days after the start of phage treatment were either completely insensitive or markedly less sensitive to all of the myophages used in the initial two cocktails (Table 2). It is difficult to speculate on the role of AbTP3phi1 in the treatment outcome, as this phage was not introduced until the end of treatment after the patient had already made considerable progress toward recovery. However, if AbTP3phi1 had been available at the start of treatment, a rational design in the phage cocktail would indicate its inclusion due to its lack of relationship to the other phages and use of a genetically independent receptor. Genomic analysis of the phages used in this intervention illustrates the importance of whole-genome sequencing of phages to be used in phage therapy, in addition to the conventional experimental tests for phage host range and growth characteristics. The use of genetically distinct phages in a phage cocktail can avoid redundancy and significantly save time and effort in phage production and purification, which is also an important consideration in making phage therapy practical. Finally, this work shows that relatively simple in vitro selections for host resistance (Fig. 5) can yield predictive results for how the organism may behave in vivo during infection.

## Methods

### Ethics statement

As required by FDA Emergency Investigational New Drug (EIND) regulations, the patient’s wife provided written informed consent prior to administration of bacteriophage therapy. The informed consent included consent to use biological specimens obtained during the course of therapy for research related to the therapeutic intervention at UC San Diego and collaborating institutions including Texas A&M University. In concordance with EIND regulations, the UC San Diego Human Research Protections Program (IORG 0000210) was notified of the EIND and provided copies of study documents within timelines outlined in the EIND regulations. The patient reviewed this manuscript and consented to the publication of personal identifiers included in the text.

### *A. baumannii* clinical isolates

As reported previously^11^, *A. baumannii* clinical isolates were isolated from multiple drains, peritoneal fluid, and respiratory secretions of the patient receiving phage treatment at the UCSD Clinical Microbiology Laboratory. Strain TP1 was isolated from peritoneal drain on Feb 10, 2016, strain TP2 and TP3 were isolated from a pancreatic drain on March 21 and March 23, 2016, respectively. All *Acinetobacter* strains were routinely cultured on tryptic soy broth (TSB, 17 g/l Bacto tryptone, 3 g/l soytone, 2.5 g/l D-glucose, 5 g/l NaCl, 2.5 g/l disodium phosphate) or Tryptic Soy Agar (TSB plus 1.5% Bacto agar, w/v). For all plaque assays, a 0.5% TB agar overlay (10 g/l tryptone, 5 g/l NaCl and 0.5% Bacto agar) was inoculated with 0.1 ml of a fresh overnight TSB culture of host and poured over TSA plates. All strains were grown at 37 °C.

### Phage propagation, whole-genome sequencing and characterization

Except for AB-Navy71, the isolation and propagation of all phages used in three cocktails, ΦPC, ΦIV, and ΦIVB were conducted using the soft agar overlay method^58^, and have been described in detail previously^11^. Phage AB-Navy71 was purchased from the Leibniz Institute DSMZ (www.dsmz.de) as phage name vB-GEC_Ab-M-G7 (DMS25639). Phage DNA was extracted using the Promega Wizard DNA extraction system following a modified protocol as described previously^59^. Phage lysates were digested with 2 µg/ml DNase I and RNase A (Sigma) for 1 h at 37 °C and precipitated in the presence of 10% PEG-8000 and 1 M NaCl at 4 °C overnight. The phage precipitate was pelleted by centrifugation at 8000 × *g*, 4 °C, 10 min and resuspended in sterile water, followed by DNA extraction using the Promega Wizard kit according to manufacturer instructions and eluting phage DNA using water heated to 80 °C. The DNA was prepared for sequencing with 550 bp inserts using a TruSeq Nano kit and sequenced as paired end 250 bp reads by Illumina MiSeq with V2 500-cycle chemistry. Reads were checked for quality using FastQC (www.bioinformatics.babraham.ac.uk/projects/fastqc) and the genome was assembled using SPAdes v3.5.0^60^. The assembled contigs were completed by running PCR amplifying the region covering the contig ends, sequencing the resulting PCR products (see Supplementary Table 1 for PCR primers used), followed by manual verification. Annotation of the assembled genome was conducted using tools in Galaxy hosted by https://cpt.tamu.edu/galaxy-pub^61^. Genes were identified using Glimmer v3^62^ and MetaGeneAnnotator v1.0^63^, and tRNAs were identified using ARAGORN v2.36^64^. The identified genes were assigned putative functions using default settings of BLAST v2.9.0 against the nr and SwissProt databases^65,66^, InterProScan v5.33^67^, and TMHMM v2.0^68^. For comparative purposes, whole-genome DNA sequence similarity was conducted using ProgressiveMauve v2.4^69^. A phylogenetic tree of the phage tail fiber proteins was constructed by aligning the protein sequences with MUSCLE v3.8^70^, and using the pipeline available at https://www.phylogeny.fr/^71^ to run the maximum likelihood analysis^72^. The tree was plotted using TreeDyn v198.3^73^. Tail fiber protein multiple sequence alignment was illustrated using Clustal Omega v1.2.2 under default settings^74^. Phage genome assembly and annotations were conducted via the CPT Galaxy and WebApollo interfaces^75–77^ under default settings (https://cpt.tamu.edu/galaxy-pub).

The seven phages described in this study and the two prophages conserved in strains TP1, TP2, and TP3 were analyzed for their relationships to species representatives of all *Caudoviricetes* phages listed in the ICTV Viral Metadata Resource 18 (Oct. 19 2021). DNA sequences for 2834 phages were retrieved from NCBI, gene prediction was performed by Prodigal 2.6.3^78^, and the results were analyzed by Gene2genome 1.1.0 to generate input files for vContact2. Analysis was conducted in vContact2 0.9.19^79^ at default settings with BLASTp comparisons, and visualized in Cytoscape 3.9.1^80^. All analyses for this comparison were conducted on the CyVerse platform^81^.

### Determination of phage sensitivity on clinical strains

Phage sensitivity of *A. baumannii* clinical isolates was determined by spotting serially diluted phage suspensions onto bacterial lawns produced by the soft agar overlay method^58^. Aliquots of 10 µl serially diluted phage were spotted onto the agar overlay plate, which was incubated at 37 °C for 18–24 h to observe plaque formation. All assays were performed in triplicate.

### Phenotype microarrays

Omnilog PM panels 1–20 for bacterial strains and Dye Mix D (Biolog, Hayward, CA) were used for phenotypic profiling of pancreatic drainage isolates TP1 and TP3. Each strain was assayed with three independent replicates for 48 h following manufacturer’s instructions. Briefly, TP1 and TP3 were grown overnight on agar plates and then suspended in OmniLog Inoculating Fluid (IF-0) and turbidity levels adjusted prior to addition of Dye Mix D in IF-10 at a 1:5 dilution and 100 µl of this suspension was pipetted into each well of the 20 PM plates with a microchannel pipettor. Plates were incubated for 48 h at 37 °C. This experiment was conducted independently three times, and then the average of the area under the curve values for each of the three replicates were analyzed using the R package opm^82^ and plotted in a heat map such that for each condition, red indicates growth inhibition, black indicates intermediate growth, and green indicates significant growth.

### Genome sequencing and genome analysis of *A. baumannii* TP1, TP2, and TP3

Genomic DNA of *A. baumannii* TP1, TP2, and TP3 was extracted using a bacterial genomic DNA extraction kit (Zymo Research) and sequenced via Illumina TruSeq and Oxford Nanopore MinIon R9 flowcell sequencing at the Texas A&M Institute for Genome Sciences and Society (TIGGS, College Station, TX). For Illumina sequencing, libraries were prepared using a TruSeq Nano kit and sequenced by Illumina MiSeq with V2 500-cycle chemistry. For Oxford Nanopore MinION sequencing, a Nanopre SQK-RAD004 Rapid Sequencing Kit was used. Illumina reads were passed through FastQ groomer^83^ and trimmed using Trimmomatic^84^ with parameter settings AVGQUAL = 25; SLIDINGWINDOW = 4, average quality required = 28; TRAILING = 25. After trimming, reads were checked for quality with FastQC (www.bioinformatics.babraham.ac.uk/projects/fastqc). For TP1, MinION reads were trimmed to remove the leading 1.5 kb and initial assembly was performed with Unicycler v0.4.8^85^ at default settings to generate a draft genome. This assembly was closed by short-read mapping with Bowtie2 v2.3.4.3^86,87^ and successive rounds of Pilon v1.20.1^88^ under default settings with variant calling mode off, followed by gap closure by resequencing of PCR products (see Supplementary Table 1 for primers used). For TP2 and TP3, untrimmed MinION reads were assembled in Canu v2.1^89^ at default settings to create a draft assembly, which was corrected by Bowtie2 mapping of Illumina reads and Pilon as above. In all assemblies, low coverage areas, areas with ambiguous base calls, and contig ends were confirmed by PCR (Supplementary Table 1). The three independently-assembled chromosomal contigs were compared in ProgressiveMauve^69^ which indicated that all assemblies were colinear without major translocations or transversions with the exception of changes in mobile elements between strains. Small SNPs and indels in TP2 and TP3 were identified by mapping Illumina reads to the TP1 chromosome in Bowtie2 (–fast mode, maximum fragment length 800). BAM files were analyzed in bcftools mpileup v1.10^90^ with max per-file depth of 250. Bcftools call v1.9 was used to identify SNPs and indels by consensus call in haploid mode with a score cutoff of 100. Analyses were conducted in Galaxy at either usegalaxy.org or usegalaxy.eu^77^. The complete genome sequences were deposited to NCBI and annotated by the NCBI Prokaryotic Genome Annotation Pipeline v5.3^91^. The closed, circular genome sequences were re-opened upstream of *dnaA*.

ARGs were identified using the CARD Resistance Gene Identifier (https://card.mcmaster.ca/) allowing for perfect and strict hits^45^ under default settings. The capsule (K) locus was identified using the Kaptive v0.7.3^32,44^. Prophage regions were detected using PHASTER^92^ and the boundaries verified by BLASTn against related bacterial genomes and identification of *attL* and *attR* sites as direct repeats. Through a workflow developed at the CPT (https://cpt.tamu.edu/galaxy-pub), the prophage regions were compared to phage and bacterial genomes available in the NCBI nt database via BLASTn^66^, and ProgressiveMauve^69^ was used to calculate percent identities. The location and size of indels and SNPs in TP2 and TP3 in reference to TP1 were determined by using ProgressiveMauve^69^, followed by manual verification.

### Generation of phage-resistant *A. baumannii* mutants in vitro

Phage-resistant mutants of *A. baumannii* TP1 were generated in vitro by spotting undiluted phage lysates (10 µl) to lawns of TP1 and picking colonies growing within the spots following overnight incubation, and streaking to fresh TSA plates. These isolates were then used to inoculate fresh TSB cultures which were grown to an OD_550_ of 0.2–0.3 and infected with the same phage at an MOI of 0.2. The cultures were incubated for 6 h at 37 °C, and then plated on TSA to produce individual colonies. A single colony was isolated from these plates and purified by an additional round of subculture. Strains were confirmed to be resistant to phage by spot assays as described above. Three independent phage-resistant mutants were isolated against phages AC4, Maestro, AB-Navy97, AbTP3phi1, and two independent mutants were isolated against phage AB-Navy1. Genetic changes in the phage-resistant mutants were determined by extraction of mutant strain DNA as described above, preparing for sequencing with an Illumina TruSeq Nano kit, and sequencing by Illumina MiSeq (V2, 500 cycles). SNPs and small indels were detected by mapping mutant Illumina reads to the parental TP1 genome as described above. Read mapping of the parental (TP1) reads against the reference genome was used to subtract spurious variant calls from mapped mutant reads, and remaining variant calls were filtered to retain calls with quality scores of 100 or greater.

### Reporting summary

Further information on research design is available in the Nature Research Reporting Summary linked to this article.

## Supplementary information

Supplementary Information

Reporting Summary

Description of Additional Supplementary Files

Supplementary Dataset 1

Supplementary Dataset 2

## Data Availability

The genomes of *A. baumannii* TP1, TP2, and TP3 were deposited in the NCBI database under BioProject PRJNA641163, with the following accession and BioSample numbers. TP1: CP056784 and SAMN15344688; TP2: CP060011 and SAMN15735522; TP3: CP060013 and SAMN15738014. Phages were deposited to NCBI under the following accession numbers: MT949699 (Maestro), OL770258 (AB-Navy1), OL770259 (AB-Navy4), OL770260 (AB-Navy71), OL770261 (AB-Navy97), OL770262 (AC4), OL770263 (AbTP3phi1). The results of CARD searches for AMR genes are provided as Supplementary Data 2.
