# Supplementary material for: Comparative genomics of Acinetobacter baumannii and therapeutic bacteriophages from a patient undergoing phage therapy
Source: Nat Commun. 2022 Jun 30;13:3776. doi: 10.1038/s41467-022-31455-5 (PMC9247103; doi:10.1038/s41467-022-31455-5)
Supplement: Supplementary file 1 — Supplementary Information [file 41467_2022_31455_MOESM1_ESM.pdf]

Supplementary Table 1. Primers used to complete the genome sequences reported in this study.

**Primers used to complete phage genome sequences**		
**Primer name**	**Primer direction**	**sequence (5' -> 3')**
C2P21/24	FWD	CTAGGACAACTGTGCGTCGCC
C2P21/24	REV	CCAGGAATTAAAGCAGTTTGCACG
AB-Navy1	FWD	GCATGTGGAGCTAATACTGAGGA
AB-Navy1	REV	GTAGAGCACTACGATGGTGACA
AB-Navy4	FWD	GCCGGATAATTTTAAACCTGCA
AB-Navy4	REV	GCACCAGTAACAGTTGCAGTAA
AB-Navy71	FWD	TTTGATTCGAAAATCAAATCGATCAAAG
AB-Navy71	REV	CCTTAATTGCTGCATTAGCTG
AB-Navy97	FWD	GAAGGCCATATTTTCTAGTGCC
AB-Navy97	REV	CCTGATAGTTCTGCGCTTAATG
AB-TP3Φ1	FWD	AGAGCGAATGCATAGAAAGAC
AB-TP3Φ1	REV	GAACATCACTCAGAGAGTGT

**Primers used to complete bacterial genome sequences**			
**TP1**			
**Primer name**	**Primer direction**	**sequence (5' -> 3')**	**location on TP1 genome**
JDC_F1_TP1_C1-2	FWD	TTGGGAACCGCTTTCACCAT	1377633
JDC_F1_TP1_C5-1	REV	GGCTGCACAGTGGTTTTCTG	1379144
TP1_gap3_FWD	FWD	TGGTCCACATACAGTGGCTG	837881
TP1_gap3_REV	REV	GCGAGATCTGTGAAAGTCGC	843014
TP1_gap5_FWD	FWD	TTGGGCTGTGATTCGTTCGT	1381828
TP1_gap5_REV	REV	CCGTGGTTCTGGGTTTTTGC	1382823
**TP2**			
**Primer name**	**Primer direction**	**sequence (5' -> 3')**	**location on TP1 genome**
TP2_end_FWD2	FWD	TTCTGGCTTCCCGCAATGAG	2492088
TP2_end_REV2	REV	GCACAAGGTAAGCGTAGAGC	2492860
TP2_FWD	FWD	ATATGCACCGCCAGACAAGG	2543120
TP2_REV	REV	AAGCGGCTGAGCAAGGAAAT	2492807
**TP3**			
**Primer name**	**Primer direction**	**sequence (5' -> 3')**	**location on TP1 genome**
TP3_end_FWD2	FWD	ACCAAGCCAACTCCCACGAA	1965094
TP3_end_REV2	REV	GGCAGCGGAAGAGCCAATTC	2024403
TP3_ends_FWD1	FWD	CACAATTAGGGGGCGTAAAAATCA	2023907
TP3_ends_REV1	REV	CATGCTCGGCCAGAATTTCG	1965873
TP3_gap1_FWD1	FWD	AGACTGAGGCTGAGGAGGAG	547097
TP3_gap1_REV1	REV	TCCAAAGCCCCTTCTGCATT	558195
TP3_gap1_REV2	REV	ATAAGCTCCTCCTGCCGTTG	547700

Supplementary Table 2. Antibiotic susceptibility of some strains collected from the patient during phage treatment.

Bacterial strains			Antibiotic sensitivity														
CPT Strain name	Date of sample collection	Source	Amikacin	Ampicillin/ Sulbactam	Cefepime	Ceftazidime	Ceftriaxone	Ciprofloxacin	Colistin	Gentamicin	Meropenem	Tetracycline	Tigecycline	Tobramycin	Doripenem	Minocycline	Trimethoprim/ Sulfamethoxazole
TP1	2/10/2016		R	ND	R	R	R	R	ND	R	R	R	ND	R	ND	ND	S
AB-SD2	3/14/2016																
Start of phage therapy: March 15th																	
AB-SD4	3/17/2016	Abscess- Drain 3	R	ND	R	R	R	R	ND	R	R	R	ND	R	ND	ND	R
AB-2392	3/17/2016		R	ND	R	R	R	R	ND	R	R	R	ND	R	ND	ND	S
AB-2399	3/17/2016	Drain 3															
AB-SD1	3/19/2016	Drain 5															
AB-SD3	3/19/2016	Drain 3															
AB-2755	3/19/2016	Drain 1	R	ND	R	R	R	R	4ug/ml	R	R	R	I	R	R	ND	R
TP2	3/21/2016	Drainage 1	I	S	ND	R	I	S	ND	R	S	R	ND	I	ND	ND	S
AB-SD5	3/21/2016	Bronchial wash															
TP3	3/23/2016	Drain 1	R	ND	R	R	R	R	ND	R	R	R	ND	R	ND	ND	R
AB-0280	3/31/2016		R	R	R	R	R	R	ND	R	R	R	ND	R	ND	ND	S
AB-1485	5/9/2016	Drain 3	R	R	R	R	R	R	2ug/ml	R	R	R	6ug/ml	R	ND	R	S
AB-3804	5/9/2016	Drain 1	R	R	R	R	R	R	4ug/ml	R	R	R	8ug/ml	R	ND	R	S
AB-3819	5/9/2016	Drain 3 (2nd sample)	R	R	R	R	R	R	2ug/ml	R	R	R	6ug/ml	R	ND	R	S
AB-3847	5/9/2016	Drain 5	R	R	R	R	R	R	4ug/ml	R	R	R	8ug/ml	R	ND	R	S
AB-0214	5/16/2016	Drain 5	R	R	R	R	R	R	ND	R	R	R	ND	R	ND	ND	S
AB-0779	5/16/2016	Drain 1	R	R	R	R	R	R	ND	R	R	R	ND	R	ND	ND	S
AB-0780	5/16/2016		R	R	R	R	R	R	ND	R	R	R	ND	R	ND	ND	S

Note: (1) Antibiotic resistance profiles were determined at the UCSD clinical laboratory. (2) Strains are determined to be either sensitive (S), resistant (R) or have an intermediate (I) phenotype. Sensitivity to some antibiotics was not determined (ND). Isolates with blank rows were not tested for AMR.

Supplementary Table 3. Locations of K locus and prophages in *A. baumannii* strains TP1, TP2, TP3.

K Locus

	start position	end position	length (bp)	genes	Best match locus	Coverage	Identity
TP1	3774031	3794556	20526	17	KL116	100%	97.4%
TP2	3780056	3800575	20520	17	KL116	100%	97.4%
TP3	3781228	3801747	20520	17	KL116	100%	97.4%

Prophages in strain TP1 (NZ_CP056784.2)*

	start position	end position	length (bp)	att sequence
Prophage 1	2671137	2723699	52563	tgaactttagggttcaagggtaacgacatgcagcggcatcttcggagcatttatttttaa
Prophage 2	2895491	2938252	42762	gtatttggtggagatggcgggagttgaac

*same prophages are identified in TP2 and TP3

Supplementary Table 4. Genomic changes in *A. baumannii* TP2 compared to TP1.

Genome_ref	Mutation type	Position in TP1	Length	Position in TP2	Mutation	TP2 Genes affected	Notes
TP1	insertion	111357	6673	111361	large IS element insertion	APH(3')-VI family aminoglycosideO-phosphotransferase, IS30-like element ISAba125 family transposase, subclass B1 metallo-beta-lactamase, bleomycin binding protein Ble-MBL, phosphoribosylanthranilate isomerase, protein-disulfide reductase DsbD N-terminal domain-containing protein, divalent-cation tolerance protein CutA, IS3-like element ISAba14 family transposase, all inserted.	8 new genes
TP1	SNP	768428	1	775105	cA, Ala to Glu	two-component system sensor histidine kinase PmrB	
TP1	SNP	848350	1	854712	aT	intergenic	
TP1	deletion	1356493	1090	1362854	IS element lost	IS30 family transposase is lost in TP2	
TP1	insertion	1381905	1845	1387177	IS element insertion	IS6-like element IS26 family transposase, aminoglycoside O-phosphotransferase APH(3')-Ia"	2 new genes
TP1	deletion	1679979	1189	1687155	IS element lost	IS4 family transposase ISAba1 is lost in TP2	
TP1	insertion	2520267	1091	2526319	IS element insertion	inserted a IS30 family transposase in TP2	
TP1	SNP	2840989	1	2848094	tC, Ile to Thr	hypothetical protein	
TP1	deletion	3143593	1094	3150708	IS element lost	IS30 family transposase is lost in TP2	
TP1	deletion	3786215	6	3792200	2 AA deleted in frame	glycosyltransferase	Same mutation found in in vitro mutants

Supplementary Table 5. Genomic changes in *A. baumannii* TP3 compared to TP1.

Genome ref	Mutation type	Position in TP1	Length	Position in TP3	Mutation	TP3 Genes affected	Notes
TP1	insertion	111357	6673	111359	large IS element insertion	ARG cassette: APH(3')-VI family aminoglycosideO-phosphotransferase, IS30-like element ISAba125 family transposase, subclass B1 metallo-beta-lactamase, bleomycin binding protein Ble-MBL, phosphoribosylanthranilate isomerase, protein-disulfide reductase DsbD N-terminal domain-containing protein, divalent-cation tolerance protein CutA, IS3-like element	8 new genes.
TP1	deletion	1356492	1090	1362845	IS element lost	IS30 family transposase is lost in TP3	
TP1	insertion	1381905	1886	1387168	IS element insertion	IS6-like element IS26 family transposase and aminoglycoside O-phosphotransferase APH(3')-Ia	2 new genes
TP1	SNP	2840989	1	2848220	tC, Ile to Thr	hypothetical protein	
TP1	insertion	2880890	1090	2888121	IS element insertion	IS30-like element ISAba125 family transposase	
TP1	deletion	3143593	1090	3151919	IS element lost	IS30 family transposase is lost in TP3	
TP1	deletion	3786204	6	3793401	2 AA deleted in frame	glycosyltransferase	Same mutation found in in vitro mutants

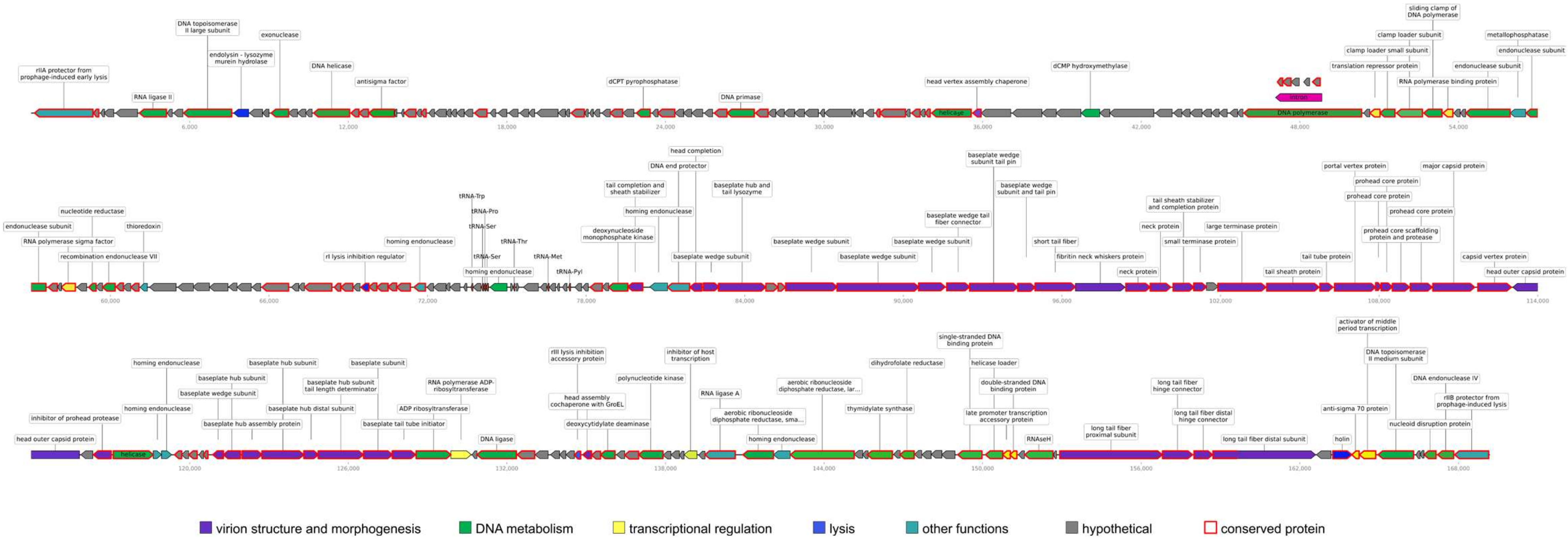

Supplementary Figure 1. Genomic map of phage Maestro. Predicted genes are represented by blocks, with blocks pointing to the right genes encoded on the forward strand, and blocks pointing to the left are on the reverse strand. The ruler below the genomes indicates scale in bp. Genes are color coded based on functions according to the legend; genes outlined in red are conserved in a set of 16 species representatives of the *Twarogvirinae*.

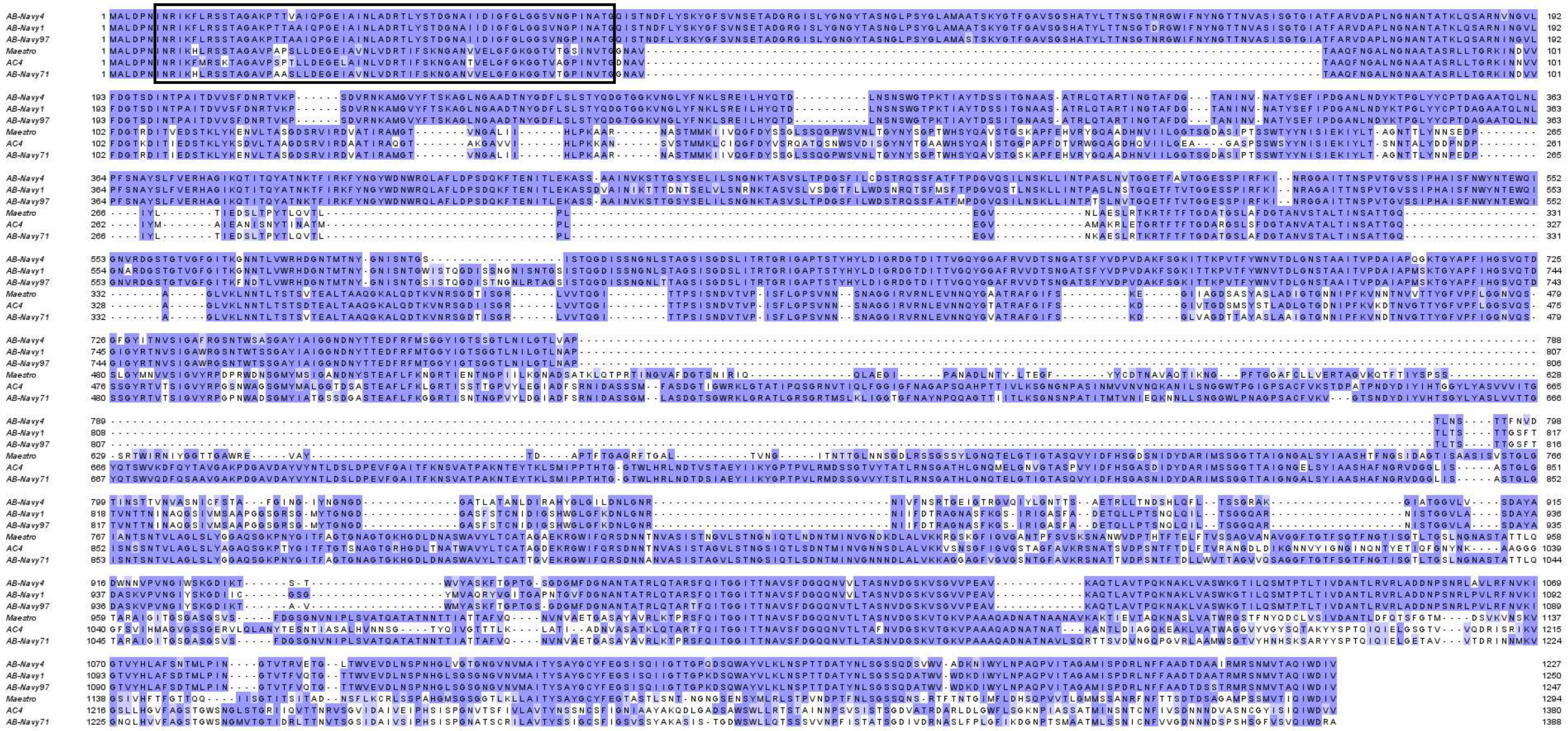

Supplementary Figure 2. Multiple sequence alignment of the long tail fiber protein sequences of the myophages used in this study. The black box denotes the regions with homology to T4 gp37.

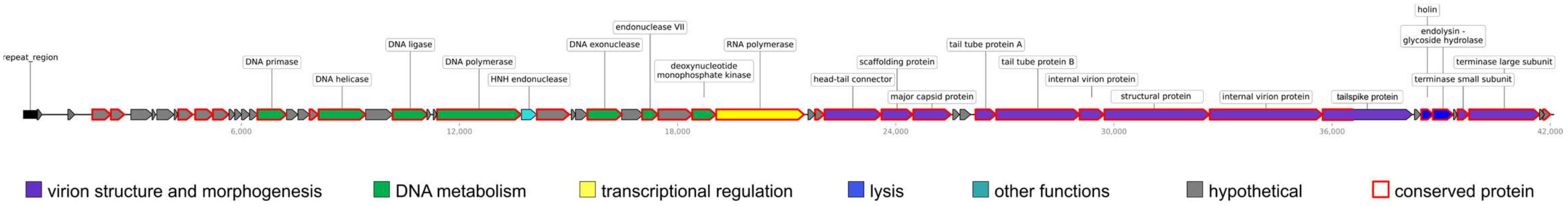

Supplementary Figure 3. Genome map of AbTP3Phi1. Predicted genes are represented by blocks, with blocks pointing to the right genes encoded on the forward strand, and blocks pointing to the left are on the reverse strand. The ruler below the genomes indicates scale in bp. Genes are color coded based on functions according to the legend; genes outlined in red are conserved in a set of 16 species representatives of the genus *Friunavirus*.
