# Supplementary material for: Comparative genomics of Acinetobacter baumannii and therapeutic bacteriophages from a patient undergoing phage therapy
Source: Nat Commun. 2022 Jun 30;13:3776. doi: 10.1038/s41467-022-31455-5 (PMC9247103; doi:10.1038/s41467-022-31455-5)
Supplement: Supplementary file 2 — Reporting Summary [file 41467_2022_31455_MOESM2_ESM.pdf]

nature portfolio

Corresponding author(s): Jason Gill

Last updated by author(s): May 28, 2022

Reporting Summary

Nature Portfolio wishes to improve the reproducibility of the work that we publish. This form provides structure for consistency and transparency in reporting. For further information on Nature Portfolio policies, see our Editorial Policies and the Editorial Policy Checklist.

Statistics

For all statistical analyses, confirm that the following items are present in the figure legend, table legend, main text, or Methods section.

n/a	Confirmed	
☐	☒	The exact sample size (*n*) for each experimental group/condition, given as a discrete number and unit of measurement
☐	☒	A statement on whether measurements were taken from distinct samples or whether the same sample was measured repeatedly
☒	☐	The statistical test(s) used AND whether they are one- or two-sided<br>*Only common tests should be described solely by name; describe more complex techniques in the Methods section.*
☒	☐	A description of all covariates tested
☒	☐	A description of any assumptions or corrections, such as tests of normality and adjustment for multiple comparisons
☒	☐	A full description of the statistical parameters including central tendency (e.g. means) or other basic estimates (e.g. regression coefficient) AND variation (e.g. standard deviation) or associated estimates of uncertainty (e.g. confidence intervals)
☒	☐	For null hypothesis testing, the test statistic (e.g. *F*, *t*, *r*) with confidence intervals, effect sizes, degrees of freedom and *P* value noted<br>*Give P values as exact values whenever suitable.*
☒	☐	For Bayesian analysis, information on the choice of priors and Markov chain Monte Carlo settings
☒	☐	For hierarchical and complex designs, identification of the appropriate level for tests and full reporting of outcomes
☒	☐	Estimates of effect sizes (e.g. Cohen's *d*, Pearson's *r*), indicating how they were calculated

Our web collection on statistics for biologists contains articles on many of the points above.

Software and code

Policy information about availability of computer code

Data collection	Illumina reads were passed through FastQ Groomer v1.1.1 and trimmed using Trimmomatic v0.36.6 with parameter settings AVGQUAL= 25; SLIDINGWINDOW = 4, average quality required = 28; TRAILING = 25. After trimming, reads were checked for quality with FastQC (www.bioinformatics.babraham.ac.uk/projects/fastqc). TP1 assembly was conducted in Unicycler v0.4.8 at default settings to generate a draft genome. This assembly was closed by short-read mapping with Bowtie2 v2.3.4.3 and successive rounds of Pilon v1.20.1 under default settings with variant calling mode off. TP2 and TP3 assembly were conducted in Canu v2.1 at default settings to create a draft assembly, which was corrected by Bowtie2 mapping of Illumina reads and Pilon as above. Phage genomes were assembled in SPAdes v3.5.0.

Data analysis	Phage genomes were annotated using Glimmer v3 and MetaGeneAnnotator v1.0 for cene calling, and tRNAs were identified using ARAGORN v2.36. The identified genes were assigned putative functions using default settings of BLAST v2.9.0 against the nr and SwissProt databases, InterProScan v5.33, and TMHMM v2.0. For comparative purposes, whole genome DNA sequence similarity was conducted using ProgressiveMauve v2.4. A phylogenetic tree of the phage tail fiber proteins was constructed by aligning the protein sequences with MUSCLE v3.8 and using the pipeline available at https://www.phylogeny.fr/ to run the maximum likelihood analysis. The tree was plotted using TreeDyn v198.3. Tail fiber protein multiple sequence alignment was illustrated using Clustal Omega v1.2.2 under default settings. Except web-based analysis, most analyses were conducted via the CPT Galaxy and WebApollo interfaces under default settings (https://cpt.tamu.edu/galaxy-pub). DNA sequences for 2,834 phages were retrieved from NCBI, gene prediction was performed by Prodigal 2.6.3, and the results were analyzed by Gene2genome 1.1.0 to generate input files for vContact2. Analysis was conducted in vContact2 0.9.19 at default settings with BLASTp comparisons, and visualized in Cytoscape 3.9.1. SNPs were identified in Bowtie2 (--fast mode, maximum fragment length 800), BAM files were analyzed in bcftools mpileup v1.10 with max per-file depth of 250. Bcftools call v1.9 was used to identify SNPs and indels by consensus call in haploid mode with a score cutoff of 100. All analyses were conducted in Galaxy at either or usegalaxy.org or usegalaxy.eu. Bacterial genomes were annotated in NCBI by the NCBI Prokaryotic Genome Annotation Pipeline (PGAP) v5.3. Antibiotic resistance genes were identified using the CARD Resistance Gene Identifier (https://card.mcmaster.ca/) allowing for perfect and strict hits. Capsule loci were identified in the Kaptive v0.7.3 at the web interface. Prophage regions were identified in the PHASTER web interface.

For manuscripts utilizing custom algorithms or software that are central to the research but not yet described in published literature, software must be made available to editors and reviewers. We strongly encourage code deposition in a community repository (e.g. GitHub). See the Nature Portfolio guidelines for submitting code & software for further information.

Data

Policy information about availability of data

All manuscripts must include a data availability statement. This statement should provide the following information, where applicable:

- Accession codes, unique identifiers, or web links for publicly available datasets
- A description of any restrictions on data availability
- For clinical datasets or third party data, please ensure that the statement adheres to our policy

The genomes of A. baumannii TP1, TP2, and TP3 were deposited in the NCBI database under BioProject PRJNA641163, with the following accession and BioSample numbers. TP1: CP056784 and SAMN15344688; TP2: CP060011 and SAMN15735522; TP3: CP060013 and SAMN15738014. Phages were deposited to NCBI under the following accession numbers: MT949699 (Maestro), OL770258 (AB-Navy1), OL770259 (AB-Navy4), OL770260 (AB-Navy71), OL770261 (AB-Navy97), OL770262 (AC4), OL770263 (AbTP3phi1).

Field-specific reporting

Please select the one below that is the best fit for your research. If you are not sure, read the appropriate sections before making your selection.

☒ Life sciences ☐ Behavioural & social sciences ☐ Ecological, evolutionary & environmental sciences

For a reference copy of the document with all sections, see nature.com/documents/nr-reporting-summary-flat.pdf

Life sciences study design

All studies must disclose on these points even when the disclosure is negative.

Sample size	Genome sequencing of three clinical bacterial isolates, seven bacteriophage isolates, 14 phage-resistant mutants. Sample size was determined based on the availability of isolates and the phages used in treatment.
Data exclusions	None
Replication	Host range studies replicated three times and presented as mean; 2-3 independent phage-resistant mutants sequenced; phenotype microarrays replicated three times and presented as mean area under the curve.
Randomization	This is an individual patient case study, randomization not possible.
Blinding	This is an individual patient case study, blinding not possible.

Reporting for specific materials, systems and methods

We require information from authors about some types of materials, experimental systems and methods used in many studies. Here, indicate whether each material, system or method listed is relevant to your study. If you are not sure if a list item applies to your research, read the appropriate section before selecting a response.

Materials & experimental systems

n/a	Involved in the study
☒	☐ Antibodies
☒	☐ Eukaryotic cell lines
☒	☐ Palaeontology and archaeology
☒	☐ Animals and other organisms
☐	☒ Human research participants
☒	☐ Clinical data
☒	☐ Dual use research of concern

Methods

n/a	Involved in the study
☒	☐ ChIP-seq
☒	☐ Flow cytometry
☒	☐ MRI-based neuroimaging

Human research participants

Policy information about studies involving human research participants

Population characteristics	All clinical specimens collected from a single 67-year-old male patient with a disseminated A. baumannii infection as part of an individual case study.
Recruitment	Patient was selected for treatment by experimental phage therapy based on condition and lack of available effective antibiotic treatments. The data presented are representative of a single patient as detailed in the manuscript.
Ethics oversight	As required by FDA Emergency Investigational New Drug (EIND) regulations, the patient's wife provided written informed consent prior to administration of bacteriophage therapy. The informed consent included consent to use biological specimens obtained during the course of therapy for research related to the therapeutic intervention at UC San Diego and collaborating institutions including Texas A&M University. In concordance with EIND regulations, the UC San Diego Human Research Protections Program (IORG 0000210) was notified of the EIND and provided copies of study documents within timelines outlined in the EIND regulations. The patient reviewed this manuscript and consented to the publication of personal identifiers included in the text.

Note that full information on the approval of the study protocol must also be provided in the manuscript.
