# Supplementary material for: Comparative genomics of Acinetobacter baumannii and therapeutic bacteriophages from a patient undergoing phage therapy
Source: Nat Commun. 2022 Jun 30;13:3776. doi: 10.1038/s41467-022-31455-5 (PMC9247103; doi:10.1038/s41467-022-31455-5)
Supplement: Supplementary file 3 — Description of Additional Supplementary Files [file 41467_2022_31455_MOESM3_ESM.pdf]

Title: Supplementary Data 1.
Description: BioLog phenotypic array plate layouts.

Title: Supplementary Data 2.
Description: Antibiotic resistance genes identified in A. baumannii strains TP1, TP2 and TP3 from the CARD database. Genes in strains TP2 and TP3 listed in red text are not present in strain TP1.
